# Interferon Lambda: Opportunities, Risks, and Uncertainties in the Fight Against HCV

**DOI:** 10.3389/fimmu.2014.00545

**Published:** 2014-10-31

**Authors:** Stephen M. Laidlaw, Lynn B. Dustin

**Affiliations:** ^1^Kennedy Institute of Rheumatology, Nuffield Department of Orthopaedics, Rheumatology, and Musculoskeletal Sciences, University of Oxford, Oxford, UK

**Keywords:** innate immunity, interferon lambda, hepatitis C virus, hepatocyte, chronic infection

## Abstract

Innate immunity is key to the fight against the daily onslaught from viruses that our bodies are subjected to. Essential to this response are the interferons (IFNs) that prime our cells to block viral pathogens. Recent evidence suggests that the Type III (λ) IFNs are intimately associated with the immune response to hepatitis C virus (HCV) infection. Genome-wide association studies have identified polymorphisms within the IFN-λ gene locus that correlate with response to IFNα-based antiviral therapy and with spontaneous clearance of HCV infection. The mechanisms for these correlations are incompletely understood. Restricted expression of the IFN-λ receptor, and the ability of IFN-λ to induce IFN-stimulated genes in HCV-infected cells, suggest potential roles for IFN-λ in HCV therapy even in this era of directly acting antivirals. This review summarizes our current understanding of the IFN-λ family and the role of λ IFNs in the natural history of HCV infection.

## Hepatitis C Virus

Hepatitis C virus (HCV) is a positive sense, single-stranded RNA virus in the family Flaviviridae. It is estimated that as many as 160 million people are chronically infected worldwide with 3–4 million new infections every year (1, 2). With a global prevalence of 2.35%, estimated to range between 0.14% on the island of Reunion and 14% in Egypt (1), there is a large economic cost and burden to society.

Transmission of HCV usually occurs following contact with infected blood through the percutaneous route, e.g., unsafe needle sharing, but may also occur through sexual transmission, iatrogenic, or vertical transmission routes. Following acute infection spontaneous resolution can occur while infected individuals that fail to clear the virus develop a chronic infection leading to liver disease, including fibrosis, cirrhosis, and hepatocellular carcinoma. Estimates of spontaneous clearance rates range from 14 to 45% depending on the population studied (3). One of the major risk factors for chronicity is HCV genotype. Currently, seven genotypes and multiple sub-types of HCV have been identified (4). Most patients are infected with only one genotype rather than multiple genotypes and within an individual the virus will mutate to form multiple genetic variants, called a quasispecies (5).

## HCV Cell Culture

*In vivo* and *vitro*, HCV primarily infects human hepatocytes. It has been possible to reconstitute the replication cycle of HCV in human non-hepatic cells such as 293T cells *in vitro* (6), and even in mice (7). Both of these approaches required the addition of extra host factors; both 293T cells and mice required the HCV entry factors claudin-1 (CLDN1) and CD81, in addition, 293T cells required occludin (OCLN), scavenger receptor class B type I (SR-BI), and the human micro RNA miR-122, while effective HCV RNA replication in mice also required the knockdown of murine innate immune mediators (7–9). Key to the replication in non-human cells seems to be the expression of the human micro RNA miR-122 (9). miR-122 has been shown to bind to the 5’ UTR of HCV and to enhance translation and replication of HCV RNA (10–14), thus, enhancing HCV propagation (15).

Experimental investigations with HCV are mainly carried out using cell-culture models. Historically, HCV was first propagated in permissive human hepatoma cell lines derived from Huh-7. Initially, only low-level replication was possible, but selection using interferon alpha (IFN-α) allowed for the isolation of cell lines that were highly permissive for replication, producing high-viral titers, including Huh-7.5 (16, 17). Key to the progression of HCV research was the development of HCV replicon systems (18). These consist of a minimal HCV genome (NS3 to NS5B non-structural genes flanked by the 5′ and 3′ UTRs) combined with a selection marker and/or reporter gene that is incapable of producing infectious virus but is capable of RNA replication *in vitro*. Another great leap occurred in 2005 when a number of groups (19–21) advanced the replicon system to show that HCV could undertake a full cycle in cell culture to produce infectious virus. This followed the identification of an HCV isolate that was replication competent without requiring tissue culture-adapting mutations (22).

Due to the limited host range of HCV, it has not been possible until recently to use small animal models of infection, and serum clinical isolates of HCV replicate poorly in tissue culture. The use of adult primary human hepatocytes (PHH) is the closest *in vitro* model of HCV infection that is currently available. Unfortunately, the availability of these is limited, resulting only from organ donation or patient biopsy. Infection efficiency of PHH is low and outcome of infection is highly variable. Even so, a number of studies are now being published using these cells (23–26).

In contrast to adult PHH, only a handful of studies have used primary fetal human hepatocytes (FHH) (27–31). These cells are long-lived and support sustained low levels of HCV replication.

## Interferons

As a positive sense single-stranded RNA virus, HCV replication necessitates the generation of double-stranded RNA intermediates (32). The infected cell identifies this as a major pathogen associated molecular pattern (PAMP), recognized by pattern recognition receptors (PRRs). These immune sensing molecules can be classified into groups, i.e., the RIG-I like receptors (RLRs), the toll-like receptors (TLRs), and the viral DNA sensors (33). Once activated these sensing molecules trigger a number of signaling pathways, resulting in the generation of Type I and III interferons (IFNs) and proinflammatory cytokines.

Identified more than 50 years ago by Alick Isaacs and Jean Lindenmann (34, 35), IFNs are the mainstay for fighting viral infections. In human beings, there are three classes of IFNs, Types I, II, and III, mainly classified on their binding to specific IFN receptors. There are multiple type I IFNs, including multiple sub-types of IFN-α, -β, -ε, -κ, and -ω, and all signal through the IFNAR complex (36). In contrast, Type II IFN comprises only one molecule, IFN-γ, which signals through the IFN-γR complex (37). Receptor binding stimulates a cascade of signal transduction events, discussed in detail below, and triggers an expression of IFN-stimulated genes (ISGs) that mediate a host of antiviral effects [reviewed by Schoggins and Rice (38)].

**Figure 1:**
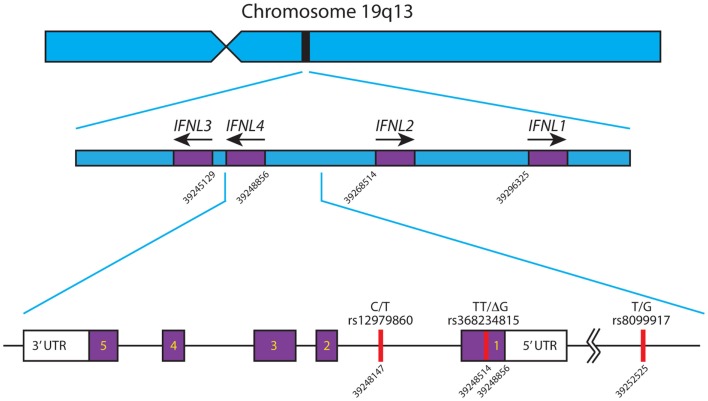
**Schematic of the IFNL gene locus**. The *IFNL1-4* genes are shown in their correct orientation and position in the region of chromosome 19 q13. Based on the human genome sequence, the nucleotide positions of the first exon of each gene are indicated. The primary SNPs, rs12979860, rs368234815, and rs8099917, affecting *IFNL3* and *IFNL4* are depicted with their corresponding nucleotide position. *IFNL4* is located on the reverse strand of chromosome 19, has 5 exons and 3′/5′ untranslated regions. All nucleotide positions are based on Ensembl genome assembly GRCh38 (December 2013), updated August 2014, database version 76 (45). Adapted from Ref. (46).

There are four known Type III IFNs, namely, IFNλ1–4 (gene names: *IFNL1–IFNL4*). These four genes are all located in a small region on the long arm of chromosome 19 (39–41) and are thought to have arisen as a result of gene duplication (42), see Figure 1. Upon the discovery of *IFNL4*, the HUGO Nomenclature Committee renamed *IL29*, *IL28A*, and *IL28B* to *IFNL1*, -*L2*, and -*L3* respectively (39–41). These cytokines signal through a heterodimeric complex consisting of the ligand-binding chain, IFN-λR1 (IL-28Rα) and the accessory chain IL-10R2 (39, 40, 43, 44).

## Restricted Expression of the IFN-λ Receptor

Whereas the Type I IFN receptor IFNAR and the IL-10R2 subunit of the IFN-λ receptor are present on virtually all human cell types, the second IFN-λ receptor subunit IFN-λR1 is expressed primarily on cells of epithelial cell origin (47) so only organs with high-epithelial cell numbers express detectable levels of IFN-λ (e.g., skin, intestine, and lungs). As expected, *in vitro* response to IFN-λ depends on the expression of IFN-λR1. In cells that lack the receptor, overexpression of IFN-λR1 can restore IFN-λ responses (48).

The greater induction of ISGs following stimulation by IFN-α compared to IFN-λ may be due in part to the number of receptors expressed by individual cells.

Human hepatic cells express both subunits of the IFN-λ receptor (IFNλ-R1 and IL10R2) (49). Addition of IFN-λ to hepatic cell lines (such as Huh-7, HepG2), Huh-7 HCV replicon expressing cell lines (49) and human primary hepatocytes (50) causes STAT1 phosphorylation and induction of ISGs, while induction was not seen following the addition of IFN-λ to primary human monocytes and lymphocytes (50) or mouse hepatocytes (51). As leukocytes and peripheral blood mononuclear cells (PBMCs) from human donors express only 6% of the level of IFNλ-R1 compared to Huh-7, this may explain their lack of response (49).

## Signal Transduction in Response to Type III IFNs

**Figure 2:**
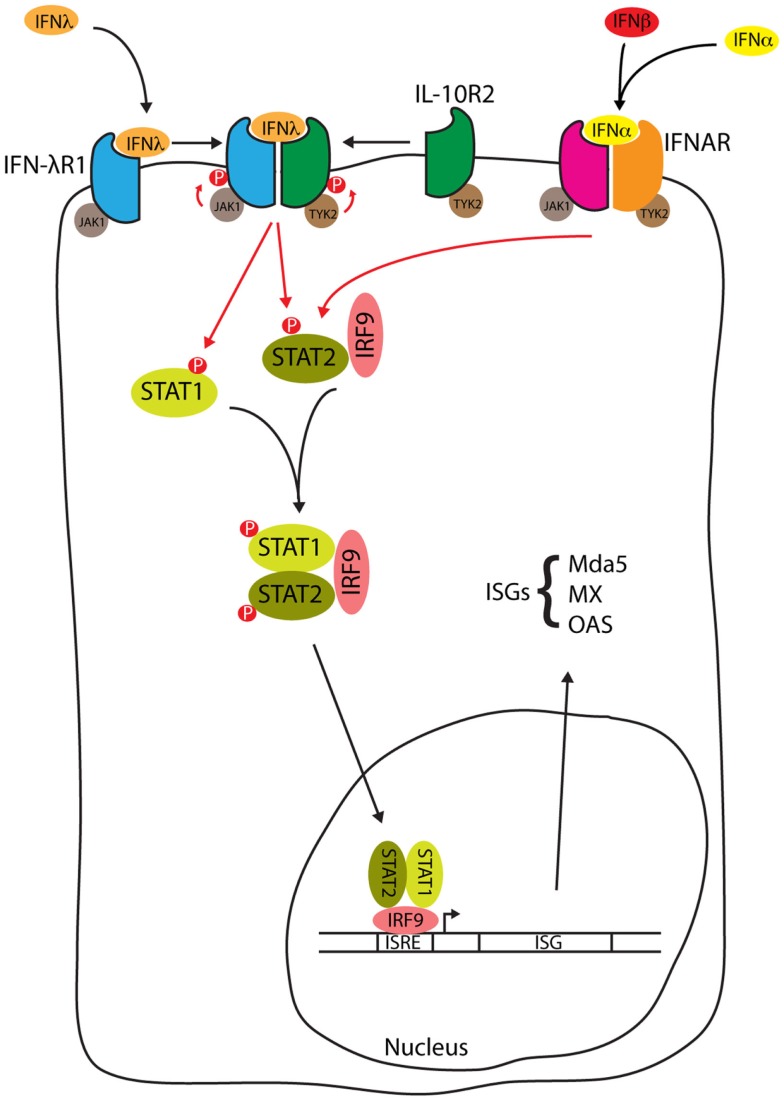
**IFN-λ signal transduction**. Type III interferon binding to the IFN-λ receptor and type I interferon binding to the IFNAR both activate the JAK1/STAT pathway to form the ISGF3 complex. ISGF3 relocates from the cytosol to the nucleus whereupon binding to ISREs induces the expression of ISGs such as myxovirus (influenza virus) resistance 1 (MX1), melanoma differentiation-associated protein 5 (MDA5), and 2′-5′ oligoadenylate synthetase (OAS), see text for details.

Signal transduction in response to Type III IFNs is similar to that seen with Type I IFN [reviewed by Au-Yeung et al. (52)], and is summarized in Figure 2. When Type III interferon binds to IFN-λR1, a conformational change allows the binding of IL-10R2 activating the receptor-associated tyrosine kinases Janus kinase 1 (JAK1) and tyrosine kinase 2 (TYK2) to cross-tyrosine-phosphorylate the IFN-λR1/IL-10R2 receptor complex allowing the recruitment of signal transducer and activator of transcription (STAT) 2 via its Src Homology 2 (SH2) domain. Further JAK tyrosine-phosphorylation of STAT2 allows the binding of the SH2 domain from cytoplasmic STAT1, resulting in the formation of a STAT1/STAT2 heterodimer. STAT2 is normally bound to IFN regulatory factor 9 (IRF9) in the cytosol, where it shuttles between the nucleus and cytosol. The addition of IRF9 to the complex forms a heterotrimeric transcription complex called IFN-stimulated gene factor 3 (ISGF3). IFN-λ may also activate STATs 3–5, although the significance of this activation has not been studied (53). Once assembled, ISGF3 relocates from the cytosol to the nucleus where it binds to interferon stimulated response elements (ISREs) upstream of ISGs [reviewed by Reich (54)], encoding proteins such as myxovirus (influenza virus) resistance 1 (MX1), melanoma differentiation-associated protein 5 (MDA5), and 2′-5′ oligoadenylate synthetase (OAS). The IFN-α and IFN-λ induction of these ISGs is reduced in STAT1 deficient hepatocytes, consistent with the requirement of STAT1 for formation of the ISGF3 complex (50). In HepG2 cells, induction of IFN-α and IFN-β is not seen when cells are stimulated with IFN-α or IFN-λ but these same cytokines do induce IFN-λ (55). Therefore, in contrast to the Type I IFNs, Type III IFNs are themselves ISGs.

Other pathways may also be activated by IFN-λ, including the AKT signaling pathway (56). In common with Type I IFN, stimulation of cells with IFN-λ leads to phosphorylation, and hence activation of the mitogen-activated protein kinase (MAPK) c-Jun N-terminal kinase (JNK), although evidence for the activation of MAPK p38 or extracellular-signal-regulated kinases (ERK) 1/2 is conflicting (48, 57) and may depend on cell type.

As Type I and Type III IFN share similar signal transduction mechanisms, the repertoire of ISGs induced upon cytokine stimulation is predicted to be similar. Most microarray analysis and RT-PCR studies have shown IFN-λ induces essentially the same genes as IFN-α in HepG2 cells, Huh-7.5 cells containing a full-length HCV replicon (FL-neo), Huh-7.5 cells, and primary mouse and human hepatocytes (50, 58–60). Of note, a study by Bauhofer et al. (61) suggests that the differentiation state of these cells may alter the repertoire of gene induction. They showed that a wider range of ISGs were induced in IFN-α or -λ treated dimethyl sulfoxide differentiated Huh-7.5 (Huh-7.5^dif^) and PHH cells, as compared to IFN-α or -λ treated wild-type Huh-7.5 cells (61).

Although most of the above-mentioned studies show similar gene expression repertoires after stimulation with IFN-α or IFN-λ, the kinetics, and magnitude of induction are different. In a study comparing global transcriptional profiles over time, Bolen et al. identified a difference in the scale of ISG induction between different cytokines in both Huh-7 cells and primary hepatocytes (62) with IFN-β > IFN-α > IFN-λ3 > IFN-λ1 > IFN-λ2. In this experiment, the same set of genes were induced with all the cytokines but whereas IFN-α stimulated gene expression wanes after approximately 6 h, IFN-β and IFN-λ1–3 stimulated gene expression continues at a high level for >24 h. The greater specific activity (ISG induction) of IFN-λ3 over other Type III IFNs as shown by Bolen et al. has also been replicated by Dellgren et al. (63) who showed that IFN-λ3 is 16-fold more active than IFN-λ2 and 2-fold more active than IFN-λ1. A recent comparison between IFN-λ3 and IFN-λ4 has shown comparable specific activity (44). One possible explanation for the differences in the scale of ISG induction between IFN-α and IFN-λ may be due to transcription factor binding differences. Chromatin immunoprecipitation assays show that less efficient remodeling at promoter sites occurs following stimulation by IFN-λ compared to IFN-α (48). Blocking signaling through the Type I receptor, IFNAR, does not abrogate the activity of IFN-λ (50). IFN-λ signaling was shown to be enhanced by HCV infection of Huh-7.5 cells, an observation, which has been attributed to up-regulation of IFN-λR1 and prolongation of JAK-STAT signaling within those cells (60).

## Expression of IFN-λ

Up-regulation of IFN-λ is seen following infection of different cells by diverse viruses such as encephalomyocarditis virus (EMCV), murine cytomegalovirus, reovirus, Sendai virus, dengue virus, and measles virus (39, 40, 64, 65), probably following RLR signaling from the peroxisome (65). Although expression of both IFN Type I and III are triggered in similar ways, differential expression can clearly be seen in organs such as the brain and central nervous system where IFN Type III expression is minimal compared to Type I IFN expression, following viral infection (47). Dendritic cells (DCs) can express IFN-λ on stimulation with double-stranded RNA and TLR3 ligand (66).

Within the liver there is an expression of both IFN-α and -λ but identification of the cell types that express IFN-λ within the infected liver is difficult to determine. It is known that freshly isolated primary FHH express IFN-λ when infected with HCV (30, 31). Until recently, it was not possible to distinguish a particular cytokine expressing cell within a population. However, in an elegant set of experiments carried out by Sheahan et al. (31), the expression profiles of individual fetal hepatocytes were determined following laser capture micro-dissection. Type III interferon induction was observed only in HCV-infected FHH cells and not by surrounding bystander cells. Furthermore IFNL1 expression was found to be dependant on HCV replication as blocking viral replication using 2′CMA (a viral replicase inhibitor) abrogated IFN-λ1 protein secretion (31).

Although there are no studies addressing the issue of IFN-λ secretion by DCs within the HCV-infected liver, it is known that DCs can secrete IFN-λ following *in vitro* stimulation (55, 66–68). As it is unlikely that DCs support significant HCV replication, exposure to the viral PAMP is thought to occur via interaction with infected hepatocytes, endocytosis of HCV virions, or exposure to viral RNA following immune cell induced death of HCV-infected cells.

There is conflicting evidence for the up-regulation of IFN-λ in HCV chronically infected liver. Some studies have shown upregulated serum levels of IFN-λ in chronic HCV patients when compared to serum levels of patients with either non-viral diseased livers or control non-diseased livers (49, 69), while others have shown lower IFN-λ serum levels in chronic HCV livers compared to non-diseased livers (70). During analysis of IFN-λ transcripts in liver biopsies, Mihm et al. showed no difference between HCV diseased liver and non-viral diseased liver although an increase in IFN-λ transcripts can be seen when HCV-infected livers were compared to healthy livers (71, 72). *In vitro* studies show that HCV infection causes IFN-λ transcription in PHH, FHH, and the human hepatocyte cell line PH5CH8 (30, 72).

## Sequence Homology of IFN-λ1–4

IFN-λ1–3 were discovered by two independent research groups following sequence analysis of the human genome (39, 40), while IFNλ-4 was discovered when genome-wide association study (GWAS) data were compared to RNA-seq analysis of PHHs treated with polyinosinic:polycytidylic acid (polyI:C), a synthetic mimic of double-stranded RNA (41).

**Figure 3:**
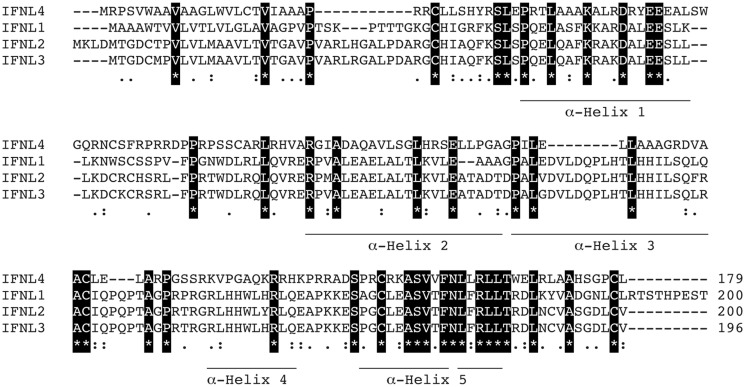
**Protein sequence analysis**. Clustal multiple sequence alignment by MUSCLE (3.8) for IFN-λs, IFNL1–4. Protein IDs are Q8IU54, Q8IZJ0, Q8IZI9, and K9M1U5-1, respectively. Identical amino-acids are shaded in black, conserved amino-acids are represented by a colon (:) and semi-conserved amino-acids by a period (.). Alpha helices are indicated and are based on the crystal structure of IFNL3 (3HHC chain A) (73–75).

IFN-λ1–4 are similar in amino-acid sequence (41–97% amino-acid conservation), especially within the first and last alpha helices, which are the primary regions of contact between Type III IFNs and their receptor, IFN-λR1 (see Figure 3; Table 1). Outside of these regions IFN-λ4 is less similar to IFN-λ1–3 having only 30% amino-acid identity. One notable region where amino-acid conservation is poor is α-helix 3, a region predicted to interact with the IFN-λ receptor subunit IL10R2 (44, 73). IFN-λ2 and IFN-λ3 are the most similar, with approximately 96% amino-acid identity (seven amino-acid differences) within their coding sequences and virtually identical within their non-coding upstream and downstream flanking sequences (41). Despite this similarity between IFN-λ2 and -3, it is remarkable that there is a 16-fold difference in specific activity (63). The reasons for this are as yet unclear, but it is interesting to note that these differences are also located in the region that is predicted to interact with the IFN-λ receptor subunit IL10R2.

**Table 1:** **Amino-acid conservation table**.

	IFNL1	IFNL2	IFNL3	IFNL4
IFNL1	100	69.5	71.9	30.7
IFNL2	*76.5*	100	96.4	26.8
IFNL3	*78.1*	*97.4*	100	29.6
IFNL4	*44.1*	*41.3*	*44.7*	100

*Clustal pairwise alignments by MUSCLE (3.8) were carried out for IFN-λs, IFNL1–4. Protein IDs are Q8IU54, Q8IZJ0, Q8IZI9, and K9M1U5-1, respectively. Percent amino-acid conservation (%) was calculated from each pairwise alignment. Identical conservation is shown in normal type and conserved conservation is shown in italic type (75)*.

Currently, two protein structures have been determined for IFN-λ; IFNλ3 (3HHC) (76) and IFNλ1 in complex with its receptor IFN-λR1 (3OG6) (77). These show that the IFN-λs exist as monomers and bind to the IFN-λR1 receptor as a 1:1 complex. The structure of IFN-λ3 shows that it is most closely related to IL-22, a member of the IL-10 family (76).

The transcription factors that regulate expression of the IFN-λ genes are currently unclear. Conflicting studies have shown requirements for IRF7 alone (IFN-α like), IRF7 plus IRF3 (IFN-β like), or IRF7, IRF3, and NF-κB (72, 78, 79). This uncertainty may be due to the wide range of cell lines used, induction protocols, and the species of IFN-λ used. Knockdown of the p65 subunit of NF-κB decreased *IFNL1* transcription in response to HCV and PolyI:C, whereas NF-κB was dispensable for the expression of *IFNL2* and *IFNL3* (72). Lee et al. suggested that this differential requirement for NF-κB could be the basis of the differential expression of the IFN-λ gene family. Recently, another transcription factor of IFNLs, Med23, was discovered to interact directly with IRF7, leading to the up-regulation of IFN-λ (80).

## IFN-λ Inhibits HCV

Studies of HepG2 cells expressing miR-122 and CD81, primary human hepatocytes, and chimpanzee and human *in vivo* studies have all shown that HCV induces primarily an IFN-λ response rather than IFN-α or -β (30, 31, 72, 81–83). IFN-λ can then act in an autocrine fashion, stimulating the expression of ISGs (30, 82). Exogenous addition of IFN-λ to hepatocytes inhibits replication of both HCV replicons (49, 58, 59, 84, 85) and cell culture-derived HCV (30, 31, 60, 70, 72, 82). In HCV replicon cell lines, recombinant IFNL1 had a greater effect on HCV replication than IFNL2 and IFNL3 (85). IFNL1 also induces STAT1 phosphorylation more readily than IFNL2/3 (49, 85), while no differences are seen between IFNL3 and IFNL4 (44). This is not the case when comparing IFN-λ activity against EMCV in HepG2 cells. Dellgren et al. (63) showed that against EMCV, IFNL3 was the most active of IFNL1–3. Concomitant with a reduction in HCV replication, IFN-λ also suppresses the microRNA miR-122 (72). The addition of both IFN-λ and the miR-122 inhibitor (miRIDIAN) increased the suppression of HCV replication (72).

## IFN-λ as a Therapy

Until recently, the standard therapy for chronic HCV was the use of pegylated interferon-α (Peg-IFN) in combination with ribavirin (RBV). Unfortunately, these regimens were poorly tolerated and often ineffective, with treatment efficacy varying among different HCV genotypes and among patients of different ethnic backgrounds or different comorbid conditions (86). Treatment with Peg-IFN and RBV results in sustained virologic response (SVR) in approximately 40–50% of genotype 1-infected people and 80% in those infected with genotypes 2 or 3 (2). HCV treatment is evolving rapidly, and current regimens for genotype 1-infected patients include either telaprevir or boceprevir, first-generation direct-acting antivirals (DAAs). These protease inhibitors (PIs) need to be administered in combination with Peg-IFN and RBV in order to reduce the likelihood of viral drug resistance. The addition of PIs leads to an increase in the likelihood of SVR to between 63 and 92% (2).

Following the discovery that the receptor for IFN-λ is cell-type restricted compared to the IFNAR receptor, it was thought that administration of IFN-λ would have fewer off-target effects and therefore patients would suffer fewer adverse side effects. As reports from *in vitro* studies suggested that HCV was sensitive to IFN-λ, there was hope that IFN-λs could be used therapeutically to combat HCV. Those hopes have largely been realized following Phase 1 trials looking at the use of pegylated IFN-λ in the treatment of genotype 1 chronic HCV infection (87, 88). The Muir et al. study compared treatment regimens consisting of Peg-IFN-λ or Peg-IFN-λ + RBV in IFN-α treatment-relapsed patients (patients who had relapsed following at least 12 weeks treatment with Peg-IFN-α + RBV) and Peg-IFN-λ + RBV in treatment-naïve patients. Although SVR was not evaluated in this study (being only 4 weeks long), the majority of patients displayed antiviral activity with a >2-log_10_ decrease in HCV RNA; in the case of treatment-naïve patients, 2 out of 7 achieved transient undetectable HCV RNA. Encouragingly, the administration of Peg-IFN-λ was well tolerated in both of the phase 1 trials with few adverse events. A possible reason for this effect with Peg-IFN-λ is that unlike IFN-α, which can produce a long-lasting refractoriness in JAK-STAT signaling (89), the use of Peg-IFN-λ induces signaling even following multiple or prolonged stimulations (90) and may also be able to overcome pre-existing refractoriness due to previous treatment with IFN-α.

Although the treatment for chronic HCV is heading toward IFN-free regimens with the development of DAAs that target either the HCV RNA polymerase (e.g., sofosbuvir) or non-structural protein NS5A (e.g., daclatasvir or ledipasvir), these treatments are costly and it is not yet known whether these treatments will be applicable to all genotypes. It may be some time until the use of IFN is discontinued, be it α or λ.

## Genome-Wide Association Studies

A number of independent groups have identified single nucleotide polymorphisms (SNPs) located near the IFN-λ3 locus that correlate with HCV treatment response and spontaneous clearance of HCV infection. These are summarized in Table 2. The polymorphisms include *rs12979860* (C/T) (91–93) and *rs8099917* (T/G) (94–97). The *rs12979860* SNP is located approximately 3 kb upstream of the IFNL3 gene, within an intron of IFNL4, while the *rs8099917* SNP is located within an intergenic region between the IFNL2 and IFNL3 genes.

**Table 2:** **SNPs at the IFNL locus**.

Variant	Genotype	Protective	Non-protective	Gene	Position in genome	Reference
rs8103142	T/C; K70R	T K70	C R70	IFNL3	39244466	(98)
rs12979860	C/T	C	T	IFNL4	39248147	(91, 92)
rs368234815	TT/ΔG	TT	ΔG	IFNL4	39248514–39248515	(41, 99)
rs8099917	T/G	T	G	5′ of IFNL4	39252525	(91, 94, 95)

*Table showing the common SNPs that have been associated with HCV clearance following either treatment with Peg-IFN + RBV or spontaneous clearance*.

These SNPs have been associated with SVR following treatment of chronic HCV with Peg-IFN and RBV (91, 94, 95, 97) and also with spontaneous clearance of HCV infection (92, 96, 100) [reviewed by O’Brien et al. (46)]. It has been shown that individuals with the non-beneficial *rs12979860*-T allele (C/T or T/T) do not respond as well to standard therapy as those individuals with two copies of the beneficial *rs12979860*-C (C/C) allele. The prevalence of the non-beneficial *rs12979860*-T allele is higher in people of African ancestry than in those of European or Asian ancestry. In a similar manner, individuals with two copies of the beneficial *rs8099917-*T (T/T) allele respond better than those individuals with one or no copies of the non-beneficial T allele (T/G or G/G) (94). As the *rs12979860* and *rs8099917* SNPs are in linkage disequilibrium (LD) with each other, favorable *rs8099917* alleles are also more prevalent in Europeans/Asians compared to Africans. Although Melis et al. did not look at patient data they have developed a method to analyze both *rs12979860* and *rs8099917* (101), which may provide greater predictability in treatment outcomes.

Recently, two groups identified a possible causal SNP upstream of the IFNL3 gene, *rs368234815* (TT or ΔG) (originally designated *ss469415590*) (41, 46, 99). This SNP results in a frameshift mutation leading to the polyI:C mediated transient expression of a new IFN-λ, now termed IFNL4 (41). The IFNL4 protein is made in IFNL4-ΔG carriers but not IFNL4-TT homozygotes. In addition, due to its location within a CpG island upstream of IFNL3, the TT/ΔG polymorphism is responsible for the methylation of a cytosine residue, which may influence gene expression (99). IFNL4-ΔG is strongly associated with impaired spontaneous clearance. In African-Americans, the IFNL4-ΔG SNP has been shown to be a better marker for predicting response following Peg-IFN and RBV than *rs12979860* (41, 102). This is in contrast to European- and Asian-Americans where the two SNPs are equally informative (41). This discrepancy is due to the differences in the degree of LD between these SNPs within these populations (99). Recently, another possible causal SNP was detected, *rs8103142* (98). This polymorphism is present within the second exon of IFNL3 and changes the amino-acid lysine at position 70 to arginine (K70R). Interestingly, this amino-acid is one of only seven that are different between IFNL2 and IFNL3 (73). Given that IFNL3 has a 16-fold greater specific activity than IFNL2, a number of groups have studied the functional consequences of this substitution *in vitro* (85, 103, 104); however, they were unable to show any difference in anti-HCV replicon activity or in the stimulation of its activity.

The molecular mechanism for the association between treatment outcome and IFN-λ SNPs is not known. The positions of these SNPs, in the vicinity of IFNL3 and IFNL4, suggest that they may be involved in transcriptional regulation although the evidence for this is conflicting. Ge et al. (91) were unable to detect a relationship between IFNL3 mRNA levels and *rs12979860* polymorphism in PBMCs from non-HCV-infected patients; Urban et al. showed that there was no significant difference in IFNL2/3 mRNA expression in HCV-infected liver tissue (103). In contrast, the presence of the non-beneficial G allele for SNP *rs8099917* was associated with lower levels of IFN-λ expression in PBMC taken from HCV-infected patients (95) and also whole blood from healthy individuals (94), but there was no significant difference in IFNL3 mRNA expression between *rs8099917* genotypes in a study carried out by Honda et al. (105). Conversely, carriers of the protective *rs12979860* C allele were shown to have higher serum protein levels of IFN-λ (70), although this may be due to LD with the IFNL4 associated SNP, *rs368234815*. In a publication by Bibert et al., PBMCs from individuals carrying different allelic combinations of *rs12979860* and *rs368234815* were stimulated with polyI:C and the expression of IFNL3 mRNA was measured. Their study showed lower expression of IFNL3 mRNA in PBMCs from individuals carrying one or two copies of the mutant ΔG allele but not by *rs12979860* (99). This study also showed that the plasma level of the ISG, IFN-γ-inducible protein 10 (IP-10), was reduced in individuals carrying the *rs368234815* non-beneficial allele but not in those carrying the *rs12979860* non-beneficial allele. Another mechanism may be the alteration of IFNL3 transcript stability via the action of HCV induced miRNA targeting of the polymorphic region of the IFNL3 3′ UTR (106).

As previously mentioned, miR-122 may play an important role in HCV replication (10). No correlation between expression of miR-122 and SNP *rs12979860* genotype was seen in human liver biopsies by Urban et al. (103) although a correlation was seen between SVR and miR-122 expression with greater expression of miR-122 in responders compared to non-responders (NR) (103, 107). In contrast, Estrabaud et al. showed that there was a significant increase of miR-122 at baseline in *rs12979860* CC genotype patients regardless of their response status (108). The authors argue that the larger numbers of patients in their study may explain this discrepancy (108). As miR-122 stimulates HCV replication *in vitro*, and antagonism of miR-122 by the oligonucleotide SPC3649 results in a decrease in serum HCV RNA *in vivo* (109), the finding that a beneficial allele may increase miR-122 is surprising. In the case of *rs12979860*, genotype C/C patients have a lower baseline ISG expression (103, 105, 110), and hence a stronger ISG activation upon infection. This innate activation may swamp any effect on HCV replication brought about by an increase in miR-122 (108).

In the case of IFNL4, the frameshift produced by the unfavorable, non-protective ΔG allele causes the production of IFN-λ4 protein. This protein was postulated to have a weak signal peptide (SP) and subsequent poor secretion (41) but subsequent experiments have shown that the SP functions correctly and that poor secretion is due to the lack of N-linked glycosylation (44). IFN-λ4 is active without glycosylation as *Escherichia coli* expressed protein is able to induce ISGs in HepG2 cells to a similar level as IFN-λ3, and addition of recombinant IFN-λ4 to Huh-7-Lunet and HepG2 cells inhibited HCV replicon replication (44). The presence of an IFN-λ4 protein that is able to induce ISG expression, is active against HCV, yet is associated with poorer HCV clearance rates is counter-intuitive. Various theories have been put forward for this effect including intracellular accumulation of non-glycosylated IFN-λ4 could be cytotoxic and result in cell death, or that IFN-λ4 could impede signaling by other IFN-λs by blocking the IFN-λ receptor and causing exhaustion of the IFN response pathways (46). IFNL4 transcripts were exclusively detected in chronic HCV liver biopsies and not in control uninfected, HBV infected, and inflammatory liver diseased liver biopsies (111), suggesting that the IFNL4 gene is specifically activated in HCV infection. In addition, Amanzada et al. were able to show a positive correlation between HCV viral loads and the amount of IFNL4 transcripts. As expected, IFNL4 transcripts were detected in all IFNL4 SNP *rs368234815* genotypes but IFN-λ4 protein can only be produced in those patients with the ΔG genotype.

It needs to be borne in mind that although GWAS studies have allowed us to narrow down the area in which a causal variant may reside, neither the identified SNPs nor the expression of the novel IFNL4 gene may be the true causal variant, and these associations may be due to some other as yet unidentified mechanism.

The IFN-λ gene region seems to have undergone genetic selection pressure, especially in the case of IFN-λ4. The negative selection of the ΔG allele and its replacement with the TT variant in non-African populations suggests that infection with another infectious agent may have driven this change. This geographical selective force probably occurred after the colonization of the New World (92). Candidates such as hepatitis B and HIV have been discounted as they do not show any association with the known IFN-λ SNPs (112–114). Also, any such disease would need to predate the movement of modern human beings out of Africa and therefore pathogens such as HIV are unlikely candidates. It remains to be seen if this polymorphism affects the outcome of other infections.

## IFN-λ and the Adaptive Immune Response

As well as its role in innate immunity, IFN-λ may have an immunomodulatory function (115). There is currently sparse literature describing the action of IFN-λ and its effect on adaptive immunity directed toward HCV, perhaps because leukocytes in general do not express the IFN-λR1 receptor (116). Though some authors have demonstrated both IFN-λR1 mRNA and cell-surface expression in T-cells, others have not been able to replicate these findings (117). It has been reported that DCs can acquire IFN-λ responsiveness following up-regulation of IFN-λR1 during their maturation from monocytes (115). If this is the case, then even though IFN-λR1 may be absent from T-cells their function may be altered by the interaction with IFN-λ stimulated DCs.

## Conclusion

Recent advances in the treatment of HCV with newly developed DAAs will revolutionize the field of HCV research but the cost of these treatments and the side effects they may engender mean that it is too soon to abandon HCV basic research. Many questions still remain to be answered, especially in HCV innate immunity and the role of IFN-λ. The identification of genetic variation at the IFNL gene locus has allowed us to begin to understand the basis of HCV resolution, but further research is required to appreciate how the IFN-λs are involved in the progression to the chronic state following acute infection. Future research should concentrate on understanding IFN-λ gene regulation and the relevance of its differential expression.

As therapy moves toward IFN-free DAA-based regimens, will the patient genotype have an effect on SVR, and if so will this SVR be affected by the same SNPs seen with IFN-α therapy or will this new therapy throw up its own set of SNPs? The development of a more relevant culture system would be advantageous for our improved understanding of HCV pathogenesis and treatment. Although the techniques of micro-patterning and 3D cell culture are advancing, these models are not yet “user friendly.” In the absence of these, greater use of physiologically relevant cells such as primary hepatocytes will allow us to tease apart the intricate pathways and interactions that need to be discovered if we are to fully appreciate the complexities of the host:HCV interaction. Researchers should not be afraid of the inherent donor liver variation; as the maxim says variety is the spice of life.

## Conflict of Interest Statement

The authors declare that the research was conducted in the absence of any commercial or financial relationships that could be construed as a potential conflict of interest.
